# Adverse pregnancy outcomes in rural Uganda (1996–2013): trends and associated factors from serial cross sectional surveys

**DOI:** 10.1186/s12884-015-0708-8

**Published:** 2015-10-29

**Authors:** Gershim Asiki, Kathy Baisley, Rob Newton, Lena Marions, Janet Seeley, Anatoli Kamali, Lars Smedman

**Affiliations:** Department of women’s and children’s Health, Karolinska Institute, Stockholm, Sweden; Medical Research Council/Uganda Virus Research Council, Uganda Research Unit on AIDS, P.O Box 49, Entebbe, Uganda; Technical Epidemiological Group, London School Hygiene and Tropical Medicine, London, UK; Department of Health Sciences, University of York, York, UK; London School Hygiene and Tropical Medicine, London, UK

**Keywords:** Adverse pregnancy outcome, Abortion, Stillbirth, Abortion rate, Stillbirth rate, Uganda

## Abstract

**Objective:**

Community based evidence on pregnancy outcomes in rural Africa is lacking yet it is needed to guide maternal and child health interventions. We estimated and compared adverse pregnancy outcomes and associated factors in rural south-western Uganda using two survey methods.

**Methods:**

Within a general population cohort, between 1996 and 2013, women aged 15–49 years were interviewed on their pregnancy outcome in the past 12 months (method 1). During 2012–13, women in the same cohort were interviewed on their lifetime experience of pregnancy outcomes (method 2). Adverse pregnancy outcome was defined as abortions or stillbirths. We used random effects logistic regression for method 1 and negative binomial regression with robust clustered standard errors for method 2 to explore factors associated with adverse outcome.

**Results:**

One third of women reported an adverse pregnancy outcome; 10.8 % (abortion = 8.4 %, stillbirth = 2.4 %) by method 1 and 8.5 % (abortion = 7.2 %, stillbirth = 1.3 %) by method 2. Abortion rates were similar (10.8 vs 10.5) per 1000 women and stillbirth rates differed (26.2 vs 13.8) per 1000 births by methods 1 and 2 respectively. Abortion risk increased with age of mother, non-attendance of antenatal care and proximity to the road. Lifetime stillbirth risk increased with age. Abortion and stillbirth risk reduced with increasing parity.

**Discussion:**

Both methods had a high level of agreement in estimating abortion rate but were markedly below national estimates. Stillbirth rate estimated by method 1 was double that estimated by method 2 but method 1 estimate was more consistent with the national estimates.

**Conclusion:**

Strategies to improve prospective community level data collection to reduce reporting biases are needed to guide maternal health interventions.

**Electronic supplementary material:**

The online version of this article (doi:10.1186/s12884-015-0708-8) contains supplementary material, which is available to authorized users.

## Background

Abortions and stillbirths are common adverse pregnancy outcomes that contribute substantially to poor maternal health. Globally, out of an estimated 210 million pregnancies, 75 million end in abortions or stillbirths [1]. In 2008, there were an estimated 43 million induced abortions worldwide and approximately half of these were unsafe because they were either carried out by a person lacking the necessary skills or in an environment that does not conform to minimal medical standards or both [2]. Almost all unsafe abortions occur in developing countries with restrictive abortion laws. For example, the unsafe abortion rate (per 1000 women aged 15-44 years) for developed countries was one compared to 16 for developing countries in 2008 [2, 3]. The East African region has the highest unsafe abortion rate (36 per 1000 women of child bearing age); the rate for Uganda is at estimated to be 54 per 1000 [3, 4]. Unsafe abortions account for up to 20 % of maternal deaths in East Africa in addition to other serious complications and disability in women [4, 5] .

Lawn et al. also reported a high correlation between stillbirths and maternal mortality; 28 countries reporting the highest stillbirth rate contributed the highest maternal mortality rate worldwide [6]. In 2009, of 2.6 million stillbirths reported globally, more than three quarters were reported from Africa and South East Asia [7].

Despite wide recognition that abortions and stillbirths are common adverse pregnancy outcomes in developing countries, reliable data are scanty in many parts of rural Africa. In the context of legal restrictions against abortions in most parts of Africa, induced abortions are under-reported. Spontaneous abortions are also under-reported because most mothers are not aware of their pregnancies in the first few weeks of gestation when spontaneous abortions are likely to occur. In most surveys no distinction is made between induced and spontaneous abortions because many women report induced abortions as spontaneous abortions [8, 9]. Most births and pregnancy losses occur outside the formal healthcare system thus making medical records unreliable for estimating pregnancy outcomes. Community based evidence on pregnancy outcome in rural Africa is therefore needed to guide interventions. The ideal approach to accurate documentation of early pregnancy losses at population level is to identify women before conception and follow them with frequent pregnancy testing until conception and then to a pregnancy outcome. Following a cohort of pregnant women from clinical recognition of pregnancy until a pregnancy outcome is another approach. These prospective approaches are costly and suffer loss to follow up [10]. Retrospective household surveys remain the most practical way to document pregnancy outcomes in low resource settings but are limited by under-reporting of outcomes. Limiting the recall period to a few years has been suggested as a way to minimise memory lapses, however, a study in Estonia showed no difference between reporting of lifetime abortion and recent abortions [11]. In contrast, analysis of data from world fertility surveys showed a higher percentage of abortions reported in surveys with recall periods less than 5 years [12].

In this study we used two survey approaches (the annual and lifetime recall) comparatively in the same population in rural Uganda to estimate the population level burden of adverse pregnancy outcomes, and examine factors associated with adverse pregnancy outcomes to inform maternal and child health programmes in Uganda.

## Methods

### Study setting

Data for this analysis are from the General Population Cohort (GPC) in Uganda. The study site is located 120 km west of the capital city, Kampala, in a rural community where demographic surveillance and medical surveys have been conducted since 1989 as described in detail elsewhere [13]. The GPC is a community-based open cohort study with approximately 22,000 residents of 25 neighbouring villages. The cohort was initially established by the UK Medical Research Council in collaboration with the Uganda Virus Research Institute to study the population dynamics of HIV transmission in rural Uganda, and now provides a platform to investigate determinants of other diseases, and health related problems focusing on maternal and child health. Agriculture is the main economic activity with rain-fed, small-holder farms for growing mainly bananas, coffee, beans, groundnuts, vegetables and a few root crops such as cassava and potatoes mainly for subsistence. Levels of education are generally low with about one third of the population attaining secondary education. Five health facilities serve the population with basic medical care, three of which offer family planning, antenatal care and deliveries. One higher level centre within the study area and a hospital 20 km away from the study area offer emergency obstetric services.

### Data collection

An annual household survey of GPC residents has been conducted since 1989, with all study village residents eligible for inclusion. Community sensitization activities precede each survey round, including local council briefings and village meetings. All households are visited by, in turn, the mapping, census and survey teams. All consenting adult residents are interviewed at home in the local language by trained survey staff and provide a blood sample for HIV testing. In selected medical surveys between 1996 and 2013, all women aged 15–49 years who had been pregnant in the last 12 months were asked specifically about the outcome of their pregnancy. In 2012–2013, additional data on life time experience of pregnancies (total number, and outcome) were collected to compare with the annual interviews (see questions in Additional file 1).

### Pregnancy outcome definitions

The World Health Organization (WHO) has defined stillbirth as foetal death late in pregnancy deferring the gestational age (GA) when a miscarriage (abortion) becomes a stillbirth to country policy [14]. In Uganda the GA cut-off for abortion and stillbirth is 28 weeks. In this paper we therefore define *Abortion* as a foetal loss before 28 weeks of gestation and *stillbirth* as a baby born with no signs of life after 28 completed weeks of gestation. *Abortion rate* is the number of abortions per 1,000 women of childbearing age and *Stillbirth rate* is the number of stillbirths per 1000 births. In this paper no distinction is made between spontaneous and induced abortions because induced abortion is illegal in Uganda and is highly stigmatized in rural communities. *Adverse pregnancy outcome* is defined as a pregnancy that did not result in a livebirth (this included both abortions and stillbirths). Age Specific Fertility Rate (ASFR) is the number of births per 1000 women in a particular age group. It is normally calculated for 5-year age groups over the reproductive ages, which are taken as 15–49 years. We also used *Total Fertility Rate* (TFR) referring to the number of live births that a woman would have had if she were subject to the current ASFR throughout the reproductive ages (15–49 years).

### Statistical analysis

Data were initially collected on paper and double entered in Microsoft Office Access, until 2009 when electronic data capture was introduced. The program contained logic programming skips and verifications that disallowed conflicting data. Stata 13 (Stata Corporation, College Station, USA) and SAS 9.4 (SAS Institute Inc., Cary, NC, USA) were used for analysis. Baseline characteristics were tabulated by study round (roughly corresponding to calendar year). Analysis of pregnancy outcomes in the past 12 months (live birth, stillbirth, abortion) and rates were examined by study round. We explored factors associated with abortion and with stillbirth in all study rounds as separate outcomes, and estimated odds ratios (OR) and 95 % CI for the associations using random effects logistic regression to account for clustering within women who reported more than one pregnancy. Age was included in all models as an *a priori* confounder. For abortions, factors whose age-adjusted association was significant at *p* < 0.10 were included in a multivariable model, and retained if they remained associated at *p* < 0.10. Because the numbers of stillbirths were small, we did not attempt to build a full multivariable model for this outcome. We also analysed pregnancy outcomes based on lifetime experience of pregnancies; computed for those who reported at least one pregnancy, the number and proportion of pregnancies ending as livebirth, abortion and stillbirth and summarised the results by age, marital status, religion education occupation, residence, phone ownership and parity. The proportion of women in the reproductive age reporting live births, stillbirths and abortions was also determined. We examined risk factors for abortions and stillbirths, as separate outcomes; the number of these events was considered as count outcome. Negative binomial regression was used to examine the effect of various risk factors on the number of abortions and stillbirths because the data were over-dispersed (variance greater than the mean); robust clustered standard errors were used to account for correlation of repeated pregnancies among women. The logarithm of the total number of pregnancies for each woman was included in the model as an offset. As with the analysis of outcomes in each round, age was considered an a priori confounder and included in all models. Factors whose age-adjusted association with the outcome was significant at *p* < 0.10 were included in a multivariable model and retained if they remained associated at *p* < 0.10. Lastly, we compared the results of two survey approaches; annual surveys between 1996 and 2013, when women were interviewed on their pregnancy experience in the preceding 12 months, versus the single survey in 2012–2013 when women were interviewed on their complete obstetric histories. This was done to evaluate the methodological biases associated with each approach.

### Ethics

The study was approved by Uganda Virus Research Institute Research and Ethics Committee and the Uganda National Council for Science and Technology. All participants were given detailed study information before a written informed consent was obtained from them.

## Results

### Results from annual surveys conducted between 1996 and 2013

#### Participant characteristics

Table 1 shows participant characteristics and pregnancy outcomes for women interviewed in each annual survey during which questions were asked about pregnancy. Overall, median age was 28 years and slightly over half were married. Between 1996 and 2013, the proportion of women with completed primary education almost doubled (from 13.3 to 20.9 %), those with education beyond primary tripled (10.0 to 36.6 %), HIV prevalence increased from 7.5 to 11.4 %, and the proportion of women reporting a pregnancy in the previous 12 months reduced from 28.2 % to 20.5 %. Antenatal attendance data were available from 2006; the proportion of women attending antenatal clinics fluctuated between 82 and 87 %, with no clear trends over the period under study.

**Table 1:** Participants characteristics and pregnancy outcome among women aged 15–49 years by study round (1996–2013)

	Round 8	Round 16	Round 18	Round 19	Round 20	Round 21	Round 22	Round 23	ALL ROUNDS
	(1996/1997)	(2004/2005)	(2006/2007)	(2007/2008)	(2008/2009)	(2009/2010)	(2010/2011)	2012/2013	1996–2013
*Women aged 15*–*49*	*N* = *1224*	*N* = *2440*	*N* = *2498*	*N* = *2450*	*N* = *2808*	*N* = *2880*	*N* = *3166*	*N* = 2766	*N* = 20100
Age group									
< 20	551 (38.7 %)	1059 (37.0 %)	990 (34.4 %)	1090 (37.2 %)	740 (26.4 %)	782 (27.2 %)	784 (24.8 %)	511 (18.5 %)	6507 (30.0 %)
20–29	408 (28.7 %)	767 (26.8 %)	769 (26.7 %)	767 (26.1 %)	838 (29.8 %)	837 (29.1 %)	896 (28.3 %)	828 (29.9 %)	6110 (28.1 %)
30–39	280 (19.7 %)	585 (20.4 %)	618 (21.5 %)	600 (20.4 %)	700 (24.9 %)	730 (25.3 %)	844 (26.7 %)	806 (29.1 %)	5163 (23.8 %)
40–49	185 (13.0 %)	454 (15.8 %)	503 (17.5 %)	477 (16.3 %)	530 (18.9 %)	531 (18.4 %)	642 (20.3 %)	621 (22.5 %)	3943 (18.2 %)
Median age (IQR) (years)	25 (19–34)	27 (19–36.5)	28 (20–37)	27 (20–37)	27 (19–36)	27 (19–36)	28 (20–37)	30 (21–39)	28 (20–37)
Marital status									
Married	–	1401 (48.9 %)	1421 (49.5 %)	1393 (47.6 %)	1551 (55.3 %)	1583 (55.0 %)	1755 (55.5 %)	1644 (59.5 %)	10748 (53.0 %)
Divorced/separated/widowed	–	393 (13.7 %)	388 (13.5 %)	390 (13.3 %)	365 (13.0 %)	385 (13.4 %)	473 (14.9 %)	448 (16.2 %)	2842 (14.0 %)
Single (never married)	–	1069 (37.3 %)	1060 (36.9 %)	1146 (39.1 %)	891 (31.7 %)	912 (31.7 %)	937 (29.6 %)	673 (24.3 %)	6688 (33.0 %)
Education									
None/less than primary	552 (38.8 %)	220 (7.7 %)	225 (7.8 %)	206 (7.0 %)	198 (7.1 %)	198 (6.9 %)	221 (7.0 %)	177 (6.4 %)	1997 (9.2 %)
Incomplete primary	539 (37.9 %)	1230 (42.9 %)	1483 (51.6 %)	1302 (44.4 %)	1026 (36.6 %)	1013 (35.2 %)	1103 (34.8 %)	998 (36.1 %)	8694 (40.0 %)
Completed primary	189 (13.3 %)	726 (25.3 %)	501 (17.4 %)	688 (23.4 %)	725 (25.8 %)	691 (24.0 %)	744 (23.5 %)	579 (20.9 %)	4843 (22.3 %)
Secondary or above	143 (10.0 %)	689 (24.0 %)	666 (23.2 %)	738 (25.2 %)	856 (30.5 %)	977 (33.9 %)	1097 (34.7 %)	1011 (36.6 %)	6177 (28.5 %)
HIV serostatus									
Positive	107 (7.5 %)	210 (7.4 %)	220 (7.7 %)	237 (8.4 %)	239 (8.6 %)	268 (9.4 %)	368 (11.9 %)	310 (11.4 %)	1959 (9.2 %)
Pregnant in last year									
Yes	400 (28.2 %)	631 (22.4 %)	643 (22.8 %)	698 (24.6 %)	757 (27.0 %)	680 (24.1 %)	736 (23.3 %)	559 (20.5 %)	5104 (23.8 %)
Attended antenatal clinic									
Yes	–	–	556 (87.1 %)	569 (83.4 %)	635 (84.2 %)	583 (85.7 %)	604 (82.4 %)	477 (85.5 %)	3424 (84.6 %)
*Women with pregnancy outcome* ^a^	*N* = *135*	*N* = *220*	*N* = *216*	*N* = *260*	*N* = *473*	*N* = *463*	*N* = *445*	*N* = *346*	*N* = *2557*
Live birth	119 (88.1 %)	179 (81.4 %)	192 (88.9 %)	218 (83.8 %)	432 (91.3 %)	431 (93.1 %)	402 (90.3 %)	309 (89.3 %)	2282 (89.2 %)
Still birth	1 (0.7 %)	7 (3.2 %)	4 (1.9 %)	14 (5.4 %)	12 (2.5 %)	7 (1.5 %)	11 (2.5 %)	5 (1.4 %)	61 (2.4 %)
Abortion	15 (11.1 %)	34 (15.5 %)	20 (9.3 %)	28 (10.8 %)	29 (6.1 %)	25 (5.4 %)	32 (7.2 %)	32 (9.2 %)	215 (8.4 %)
*Any adverse pregnancy outcome* ^*b*^	*16 (11.9 %)*	*41 (18.6 %)*	*24 (11.1 %)*	*42 (16.2 %)*	*41 (8.7 %)*	*32 (6.9 %)*	*43 (9.7 %)*	*37 (10.7 %)*	*276 (10.8 %)*
Rates									
Abortion rate (per 1000 women) ^c^	12.2	13.9	8.0	11.4	10.3	8.7	10.1	12.5	10.5
Stillbirth rate (per 1000 births) ^d^	8.4	37.6	20.5	60.3	27.0	16.0	26.6	16.5	26.2
Birth rate per 1000 women^e^	96.4	73.4	76.5	89.0	153.8	149.7	127.0	112.8	112.8
Total fertility rate^f^	3.1	2.5	2.5	2.9	5.2	5.2	4.4	4.5	3.8

^a^Women who report a pregnancy in past 12 months who are not still pregnant. ^b^Stillbirth, miscarriage or termination. ^c^Number of abortions per 1000 women of reproductive age (15–49 years). ^d^Number of stillbirths per 1000 births (live births + stillbirths). ^e^Number of births per 1000 women of reproductive age (15–49 years). ^f^Number of births that each woman would have if she were subject to the current age-specific fertility rates (ASFR) throughout her reproductive years (15–49 years). ASFR calculated as [number of births]/[number of women] in each 5-year age band; total fertility rate estimated as ∑(ASFR in each 5-year age band) * 5

#### Pregnancy outcomes and trends

Overall, 1800 women aged 15–49 reported a total of 2558 pregnancy outcomes in the previous 12 months within the study period 1996–2013. Among these, 276 women had at least one adverse outcome, 21 women had two adverse pregnancy outcomes, and one woman had three adverse pregnancy outcomes. Of the pregnancy outcomes reported in each survey, 81–93 % were a livebirth, 1–5 % were stillbirth and 5–15.5 % were abortion, resulting in an overall proportion of adverse pregnancy outcomes of 10.8 % across all the surveys (abortion 8.4 % and stillbirth 2.4 %). There was some fluctuation in the proportion of pregnancies with adverse outcomes with increases in the years 2004/2005 and 2007/2008. During the entire period 1996–2013, there were no consistent trends in the abortion rate but stillbirth rates doubled in the same period with considerable variation between each year. The total fertility rates increased from approximately 3 to 5 over the same period (Table 1). The overall abortion rate was 10.5 per 1000 women of reproductive age and stillbirth rate was 26.2/1000 births.

### Results from the complete obstetric histories conducted in 2012–2013

During the survey round conducted in 2012–2013, a total of 2657 women aged 15–49 years were approached for interview, 332 had missing data on pregnancy, leaving 2325 with data on lifetime pregnancy,167 of whom had never been pregnant. Of those who reported a pregnancy (2158), 569 (26.4 %) reported at least one abortion and 120 (5.6 %) reported at least one stillbirth resulting in one third of women reporting an adverse outcome. In total 11,532 pregnancies were reported by the women over their lifetime with 11,477 outcomes; 10500 (91.5 %) as a livebirth, 830 (7.2 %) as abortion and 147 (1.3 %) as stillbirth. The overall proportion of lifetime adverse pregnancy outcomes was 8.5 %. The abortion rate was 10.5 per 1000 women of reproductive age and stillbirth rate was 13.8 per 1000 total births; the total fertility rate was 4.5.

#### Lifetime adverse pregnancy outcome distribution by background characteristics

**Fig. 1:**
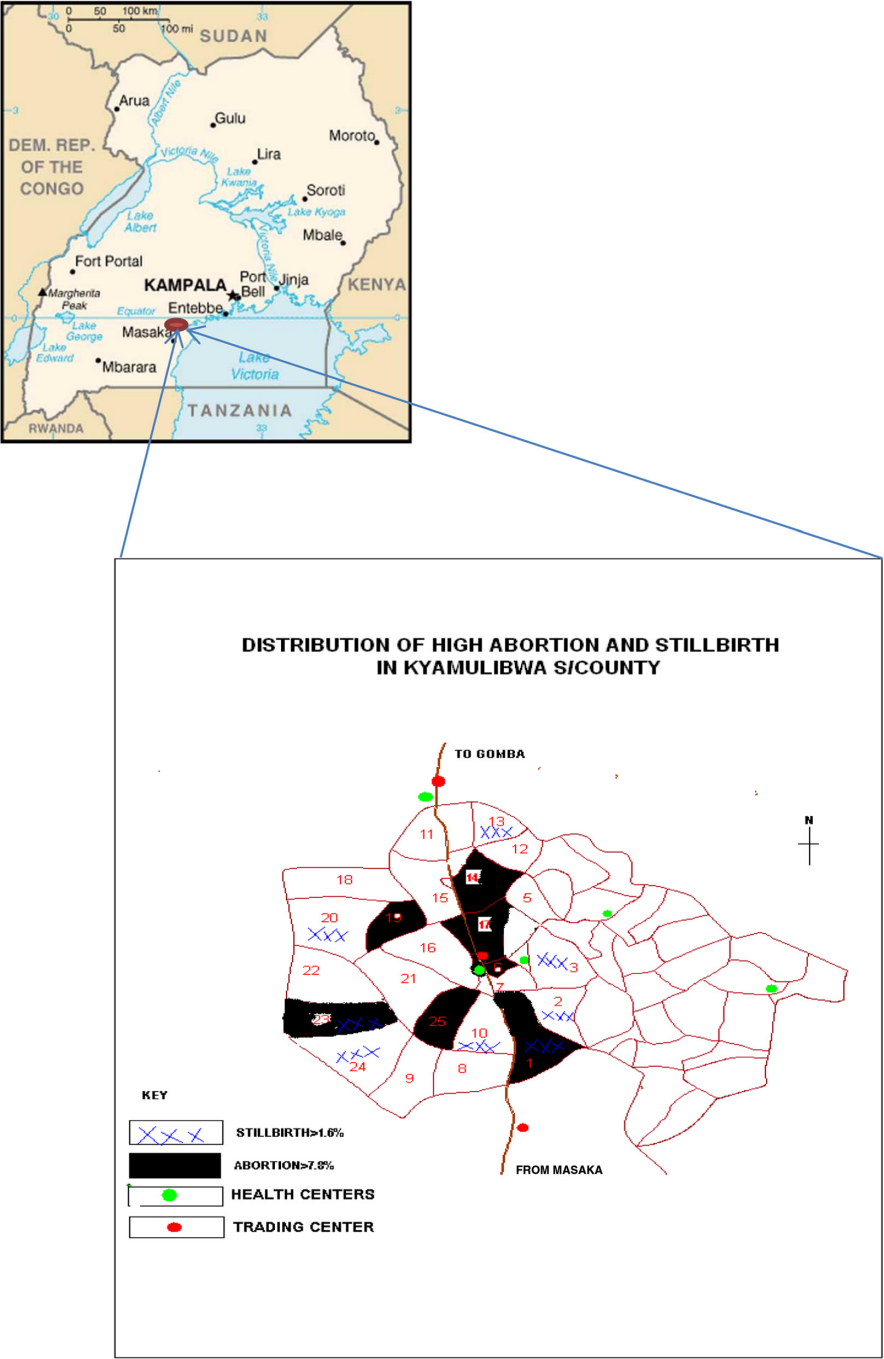
Map of Uganda showing study area and villages with high adverse pregnancy outcomes

There were wide variations in the pattern of distribution of lifetime abortions (range = 4.3–11.1 %) and stillbirths (range = 0.2–3.8 %) across the 25 villages in the study area (Fig. 1). Whereas for abortions, villages in the upper quartile were along the main road to the regional town, those with stillbirths in the upper quartile are at the periphery of the study area bordering non-study villages.

The distribution of lifetime adverse pregnancy outcomes by other characteristics is shown in Table 2. The proportion of lifetime abortions and stillbirths followed a U shaped distribution with age, highest in women under 20 years and those aged 45–49 years. The proportion of abortions increased with age at first pregnancy and the same pattern was observed for stillbirths.

**Table 2:** Distribution of pregnancies and outcomes by background characteristics among women aged 15–49 years, who reported lifetime pregnancy outcomes in 2012-2013

	Number of Women (%)	Number of Pregnancies	Pregnancy outcome (%)		
Background characteristic	(*N* = 2158)	(*N* = 11,477)	Live birth	Abortion	Stillbirth
Current Age (years)					
less than 20	121 (5.6)	153	88.9	9.2	2.0
20–29	692 (32.1)	2023	92.7	6.4	0.9
30–39	771 (35.7)	4606	91.5	7.3	1.2
40–49	574 (26.6)	4695	91.0	7.5	1.5
Age at first pregnancy (years)					
Under 15	87 (4.0)	639	91.2	8.5	0.3
15–19	1517 (70.4)	8501	91.6	7.1	1.3
20–24	483 (22.4)	2086	91.5	7.0	1.5
25–29	61 (2.8)	209	86.6	11.0	2.4
30–34	6 (0.3)	27	81.5	18.5	0.0
Ever married					
Yes	1969 (91.2)	11070	91.5	7.3	1.2
No	189 (8.86.7)	407	91.2	6.6	2.2
Tribe					
Muganda	1465 (73.3)	7955	91.4	7.3	1.3
Other tribe	533 (26.7)	2862	91.8	7.0	1.3
Religion					
Roman catholic	1176 (58.9)	6175	91.5	7.3	1.1
Anglican	231 (11.6)	1299	91.5	7.9	0.5
Muslim	486 (24.3)	2767	91.5	6.7	1.7
Others	105 (5.3)	576	90.3	6.4	3.3
Education level attained					
None/less than primary	157 (7.3)	1130	91.8	6.6	1.6
Incomplete primary	873 (40.5)	5329	91.3	7.3	1.3
Complete primary	489 (22.7)	2556	92.8	6.0	1.3
Secondary and above	639 (29.6)	2462	90.3	8.6	1.1
Village of residence					
Away from main road	1637 (75.9)	8783	92.1	6.6	1.2
Along the main road	521(24.9)	2694	89.3	9.1	1.5
Phone access					
No access to a phone	1225 (57.1)	6432	91.4	7.2	1.4
Owns a phone	677 (31.5)	3805	91.1	7.6	1.3
Family member/Neighbour’s	245 (11.4)	1173	93.0	6.6	0.4
Parity					
0–4	1051(48.7)	2805	87.9	10.0	2.1
5–9	940 (43.6)	6699	92.5	6.4	1.1
≥10	167 (7.7)	1973	93.0	6.2	0.8
Antenatal attendance last 1 year^a^					
Yes	463 (85.1)	2087	90.6	8.2	1.2
No	81 (14.9)	339	81.1	18.0	0.9
HIV status					
Infected	293 (13.8)	1501	91.0	7.9	1.1
Uninfected	1860 (86.2)	9975	91.6	7.1	1.3

^a^Only those who reported pregnancy in the past 12 months were asked on atleast one episode of antenatal attendance and therefore 1614 had missing data on antenatal care. There was also missing data for age at first pregnancy for four women, missing data on tribe for 160 women, missing data on religion for 160 women, missing data on phone ownership for 11 women, missing data on HIV status for one woman. The number of pregnancies in these categories with missing data does not therefore add up to the total pregnancies (11,477)

Women who had attained education above secondary reported slightly more lifetime abortions compared to those who had attained less education but the reverse is true for stillbirths. Both abortion and stillbirth proportions reduced with increasing parity. As expected, non-attendance of antenatal care for a pregnancy in the past 12 months was associated with higher lifetime abortion frequency but this was not the case for stillbirths. There were small differences in lifetime adverse pregnancy outcomes by HIV status, marital status, tribe, and phone ownership.

#### Factors associated with lifetime pregnancy outcomes (Unadjusted and Adjusted analysis)

In the unadjusted analysis, lifetime abortion risk increased with higher education, non-attendance of antenatal clinic, proximity to the main road and was less among those with higher parity (Table 3). In the final model the association with parity, village of residence and antenatal care remained significant and abortion risk increased with increasing age. There was some evidence suggesting that a higher level of education were associated with an increase in abortion risk, and not being married with less risk of abortion. Lifetime stillbirth risk increased with age and reduced with increasing parity in the adjusted analysis (Table 4).

**Table 3:** Factors associated with lifetime abortions, among women interviewed about their lifetime experience of pregnancies in 2012–2013, rural south- western Uganda

	n abortions / N pregnancies (%)	Unadjusted RR (95 % CI)	Adjusted RR (95 % CI)^a^
Age group		*P* = 0.44	*P* < 0.001
<20	14 / 153 (9.2 %)	1	1
20–29	129 / 2023 (6.4 %)	0.76 (0.44–1.32)	0.84 (0.49–1.45)
30–39	335 / 4606 (7.3 %)	0.87 (0.50–1.50)	1.36 (0.78–2.35)
40–49	352 / 4695 (7.5 %)	0.89 (0.52–1.53)	1.70 (0.97–2.97)
Ever married		*P* = 0.71	*P* = 0.09
Yes	803 / 1170 (7.3 %)	1	1
No	27 / 407 (6.6 %)	0.93 (0.64–1.35)	0.73 (0.51–1.05)
Education		*P* = 0.03	*P* = 0.07
None/less than primary	75 / 1130 (6.6 %)	1	1
Incomplete primary	390 / 5329 (7.3 %)	1.09 (0.83–1.44)	1.08 (0.82–1.42)
Completed primary	153 / 2556 (6.0 %)	0.90 (0.66–1.23)	0.84 (0.61–1.14)
Secondary or above	212 / 2462 (8.6 %)	1.28 (0.95–1.73)	1.11 (0.82–1.50)
Village location		*P* < 0.001	*P* < 0.001
Away from main road	584 / 8783 (6.6 %)	1	1
Along the main road	246 / 2694 (9.1 %)	1.37 (1.15–1.63)	1.33 (1.12–1.57)
Parity		*P* < 0.001	*P* < 0.001
0–4	280 / 2805 (10.0 %)	1	1
5–9	428 / 6699 (6.4 %)	0.66 (0.56–0.78)	0.50 (0.40–0.61)
≥10	122/ 1973 (6.2 %)	0.63 (0.49–0.81)	0.43 (0.31–0.59)
Attended antenatal clinic^b^		*P* < 0.001	*P* < 0.001
Yes	172 / 2087 (8.2 %)	1	1
No	61 / 339 (18.0 %)	2.15 (1.60–2.90)	2.14 (1.60–2.86)

^a^Adjusted for age, ever married, education, village location and parity. ^b^Attended antenatal care data restricted to women reporting a pregnancy in the past 12 months. Restricted to women reporting a pregnancy in the past 12 months

**Table 4:** Factors associated with lifetime stillbirths, among women interviewed about their lifetime experience of pregnancies in 2012–2013, rural south- western Uganda

	n stillbirths / N pregnancies (%)	Unadjusted RR (95 % CI)	Adjusted RR (95 % CI)^a^
Age group		*P* = 0.27	*P* < 0.001
<30	21 / 2209 (1.0 %)	1	1
30–39	55 / 4589 (1.2 %)	1.25 (0.71–2.19)	2.21 (1.21–4.03)
40–49	71 / 4669 (1.5 %)	1.58 (0.89–2.80)	3.60 (1.84–7.04)
Ever married		*P* = 0.11	*P* = 0.44
Yes	138 / 11055 (1.2 %)	1	1
No	9 / 412 (2.2 %)	1.85 (0.87–3.94)	1.35 (0.63–2.89)
Education		*P* = 0.72	*P* = 0.29
None/less than primary	18 / 1128 (1.6 %)	1	1
Incomplete primary	71 / 5308 (1.3 %)	0.88 (0.36–2.11)	1.05 (0.55–1.99)
Completed primary	32 / 2551 (1.3 %)	0.82 (0.33–2.04)	0.91 (0.45–1.83)
Secondary or above	26 / 2480 (1.0 %)	0.66 (0.26–1.70)	0.63 (0.29–1.34)
Village location		*P* = 0.30	*P* = 0.33
Away from main road	106 / 8767 (1.2 %)	1	1
Along the main road	41 / 2700 (1.5 %)	1.26 (0.81–1.96)	1.23 (0.81–1.86)
Parity		*P* = 0.004	*P* < 0.001
0–4	59 / 2839 (2.1 %)	1	1
5–9	72 / 6683 (1.1 %)	0.55 (0.36–0.82)	0.35 (0.22–0.56)
≥ 10	16 / 1945 (0.8 %)	0.42 (0.22–0.80)	0.21 (0.10–0.43)
Attended antenatal clinic^b^		*P* = 0.64	*P* = 0.48
Yes	25 / 2099 (1.2 %)	1	1
No	3 / 361 (0.8 %)	0.74 (0.22–2.55)	0.65 (0.19–2.15)

^a^Adjusted for age, ever married, education, village location and parity. ^b^Attended antenatal care for a pregnancy in the past 12 months. Restricted to 537 women reporting a pregnancy in the past 12 months

For the annual reporting of pregnancy outcomes, only older age, non- attendance of antenatal care and more recent year of survey were associated with abortions (Additional file 2). There was some evidence of lower odds of reporting stillbirths ten years before the latest survey (Additional file 3).

## Discussion

One third of women aged 15–49 in rural south-western Uganda reported at least one adverse pregnancy outcome during their lifetime. One in ten of the pregnancies reported were lost as abortions or stillbirths. The overall abortion rate was 10.8 per 1000 women of reproductive age and stillbirth rate was 26.2 per 1000 births by annual survey method. The complete obstetric histories obtained in one interview yielded a similar abortion rate (10.5/1000) but only a half of stillbirth rate (13.8/1000) compared to the annual survey method.

A national survey of health facilities in Uganda recorded more than five-fold higher abortion rate (54/1000), with one fifth of pregnancies lost as abortions [5]. The health facility survey findings are in agreement with findings from a prospective cohort studies in rural Ethiopia and rural India that reported 25 % of pregnancies lost as abortions and also in agreement with global estimates [2, 15, 16]. It is therefore evident that abortions have been grossly under-reported in our study possibly because induced abortion is illegal, and highly stigmatised in Uganda as observed by Moore and colleagues [17].

In contrast, our stillbirth rate derived from the annual survey is consistent with the national estimate (26.2 vs. 25.0 per 1000 births) but slightly higher than the stillbirth rate of 19 per 1000 births reported from a prospective community based study in rural Eastern Uganda [18], and double that we found through life time survey approach.

We observed a negligible change in the trend of adverse pregnancy outcome from 1996 to 2013 which is in line with the relatively constant abortion rates reported in the African region [2]. In contrast to reports showing a decline in stillbirth rates by 14 % globally, and by 8 % in Africa between 1995 and 2009 [19], our study showed an increase in stillbirth rate. This may be attributed to poor access to quality maternal health services in rural areas in Uganda. The most recent Uganda Demographic and Health Survey showed that only 48 % of pregnant mothers complete the four recommended antenatal visits and only 40 % give birth in health facilities in rural southwestern Uganda [5]. In low and middle-income countries, about one-third of stillbirths occur during labour as a result of prolonged labour or obstructed labour not attended to promptly [20]. Although we did not specifically quantify stillbirths at labour in our study it is possible that a number of the stillbirths reported could have occured during labour at home or due to delayed access to health facilities. Another possible reason for the apparent rise in stillbirth rate in the later years could be that women were more willing to report these events after building some trust with the study teams.

When we explored factors associated with adverse pregnancy outcome, it was not surprising to find a positive correlation with lack of antenatal attendance since antenatal care offers timely screening of pregnancy risks to prevent complications. Other factors that we found to be associated with adverse pregnancy outcomes such as age of mother and parity are in agreement with findings elsewhere [21, 22]. There is evidence suggesting that at older maternal age, risk of other diseases is cumulative and genetic defects become more common leading to spontaneous abortions. The effect of village of residence may be related to importance of access to resources, ability to access abortion services and prevailing socio-economic inequalities as observed earlier [23, 24].

Our study had some limitations. We could not distinguish between induced and spontaneous abortion as earlier reported by Rogo about surveys in Africa [25]. A number of mothers could have purposively concealed information on induced abortions as noted earlier by Barreto [26]. Besides the purposive concealment of abortions, both survey approaches are also subject to under-reporting due to forgetting of pregnancy events. Castinello and colleagues also reported in world fertility surveys in developing countries that retrospective surveys estimate only about 50–80 % of actual pregnancy losses [12]. In comparing the two approaches, we found a higher proportion of adverse pregnancy outcome from annual surveys than in the snapshot survey of complete obstetric histories due to the differences in reporting stillbirths. There are two possible reasons; first, the shorter recall period in the annual surveys enabled mothers to remember more of their pregnancy experiences than in the lifetime experience. Secondly, a sampling probability bias in the annual survey method favours adverse pregnancy outcome reporting. As observed by Weinberg et al., mothers who had an adverse outcome in the previous 12 months were more likely to be sampled for the interviews than those who had a livebirth because of a shorter interval from occurrence of outcome to interview date [27]. Another possible source of bias is the misclassification of abortions and stillbirths due to uncertainty of gestation age by most mothers in rural communities. Additionally, stillbirths may not be easily distinguished from early neonatal deaths in a rural population where half of the mothers deliver at home.

However, the large sample size coupled with several rounds of survey has enabled us to assess a number of possible determinants and trends of adverse pregnancy outcomes. We also had the opportunity to compare different approaches in household surveys and the extent to which they estimate adverse pregnancy outcomes. There is a high level of agreement in the estimates of abortion rate and total fertility rate by the two methods. Our stillbirth rate estimates by the 12 month recall seems more plausible as it compares well with estimates by other methods in Uganda [5, 18].

## Conclusion

Collecting population level data on adverse pregnancy outcome through different survey approaches is a challenge especially in settings where induced abortions are illegal and this may be an obstacle to public health action. Despite under-reporting, we found up to one third of mothers experiencing an adverse pregnancy outcome in this rural population. Key strategies for promoting uptake of antenatal services are needed to improve the outcomes of pregnancy. Qualitative studies to understand in depth the magnitude of stigma for abortions and referral pathways for care and local practices regarding induced abortion in rural Uganda will be required to guide policy action. One possible strategy to reduce adverse pregnancy outcomes at the community level is to empower community health workers to register and follow pregnant mothers through household visits to promote uptake antenatal care and facility delivery. Continued family planning promotion to prevent unwanted pregnancies should be one of the key priorities in reducing abortion rates.

## Additional files

Additional file 1:
**Questions asked on pregnancy and outcomes in each of the study rounds.** (DOCX 15 kb) Additional file 2:
**Factors associated with abortion, among 2557 women aged 15–49 years reporting a pregnancy in the past 12 months (1996–2013).** (DOCX 17 kb) Additional file 3:
**Factors associated with stillbirth, among women aged 15–49 years reporting a pregnancy in the past 12 months (1996–2013).** (DOCX 15 kb)
