# Supplementary material for: Adverse pregnancy outcomes in rural Uganda (1996–2013): trends and associated factors from serial cross sectional surveys
Source: BMC Pregnancy Childbirth. 2015 Oct 29;15:279. doi: 10.1186/s12884-015-0708-8 (PMC4627380; doi:10.1186/s12884-015-0708-8)
Supplement: Additional file 1: — Questions asked on pregnancy and outcomes in each of the study rounds. (DOCX 15 kb) [file 12884_2015_708_MOESM1_ESM.docx]

**Additional file 1: Questions asked on pregnancy and outcomes in each of the study rounds**

| **Questions asked in the study rounds** | **Study rounds** | | | | | | | |
| --- | --- | --- | --- | --- | --- | --- | --- | --- |
|  | **8** | **16** | **18** | **19** | **20** | **21** | **22** | **23** |
| Have you been pregnant in the last 12 months | √ | √ | √ | √ | √ | √ | √ | √ |
| What was the outcome of pregnancy in the last 12 months | √ | √ | √ | √ | √ | √ | √ | √ |
| How many times have you ever become pregnant, including abortions/miscarriages/stillbirths? |  |  |  |  |  |  |  | √ |
| How many of the children were born alive? |  |  |  |  |  |  |  | √ |
| How many were stillbirths? |  |  |  |  |  |  |  | √ |
| How many were abortions? |  |  |  |  |  |  |  | √ |
| In the past 12 months, how many times have you become pregnant? |  |  |  |  |  |  |  | √ |
| What was the outcome of each pregnancy in the last 12 months? |  |  |  |  |  |  |  | √ |
| When did the miscarriage or abortion occur? |  |  |  |  |  |  |  | √ |
| When did the stillbirth occur? |  |  |  |  |  |  |  | √ |
| When was the baby (were the babies) born? |  |  |  |  |  |  |  | √ |
