# Supplementary material for: Adverse pregnancy outcomes in rural Uganda (1996–2013): trends and associated factors from serial cross sectional surveys
Source: BMC Pregnancy Childbirth. 2015 Oct 29;15:279. doi: 10.1186/s12884-015-0708-8 (PMC4627380; doi:10.1186/s12884-015-0708-8)
Supplement: Additional file 2: — Factors associated with abortion, among 2557 women aged 15–49 years reporting a pregnancy in the past 12 months (1996–2013). (DOCX 17 kb) [file 12884_2015_708_MOESM2_ESM.docx]

**Additional file 2: Factors associated with abortion, among 2557 women aged 15-49 years reporting a pregnancy in the past 12 months (1996-2013)**

|  | **n abortions / N pregnancies (%)** | **Unadjusted OR (95% CI)** | **Age-adjusted OR (95% CI)** | **Adjusted OR  (95% CI)^1^** |
| --- | --- | --- | --- | --- |
| **Age group** |  | P<0.001 | P<0.001 | P<0.001 |
| <20 | 17 / 339 (5.0 %) | 1 | 1 | 1 |
| 20–29 | 76 / 1272 (6.0 %) | 1.23 (0.70 -2.15 ) | 1.23 (0.70 -2.15 ) | 1.47 (0.67 -3.23 ) |
| 30–39 | 74 / 799 (9.3 %) | 1.98 (1.12 -3.49 ) | 1.98 (1.12 -3.49 ) | 2.85 (1.27 -6.42 ) |
| 40–49 | 48 / 147 (32.7%) | 10.51 (5.33 -20.73) | 10.51 (5.33 -20.73) | 12.07 (4.56 -32.00) |
| **Marital status^2^** |  | P=0.003 | P=0.06 | P=0.21 |
| Married | 159 / 2034 (7.8 %) | 1 | 1 | 1 |
| Divorced/separated/widowed | 28 / 179 (15.6%) | 2.34 (1.40 -3.91 ) | 1.84 (1.12 -3.01 ) | 1.85 (0.94 -3.64 ) |
| Single (never married) | 13 / 207 (6.3 %) | 0.78 (0.41 -1.48 ) | 1.19 (0.63 -2.25 ) | 1.20 (0.55 -2.62 ) |
| **Education^2^** |  | P=0.08 | P=0.28 | P=0.27 |
| None/less than primary | 23 / 169 (13.6%) | 1 | 1 | 1 |
| Incomplete primary | 83 / 1006 (8.3 %) | 0.52 (0.29 -0.94 ) | 0.64 (0.37 -1.13 ) | 0.72 (0.31 -1.67 ) |
| Completed primary | 48 / 654 (7.3 %) | 0.43 (0.22 -0.81 ) | 0.61 (0.33 -1.12 ) | 0.96 (0.40 -2.32 ) |
| Secondary or above | 61 / 727 (8.4 %) | 0.53 (0.28 -0.97 ) | 0.80 (0.45 -1.43 ) | 1.21 (0.51 -2.85 ) |
| **HIV serostatus^2^** |  | P=0.13 | P=0.18 | P=0.50 |
| Negative | 188 / 2304 (8.2 %) | 1 | 1 | 1 |
| Positivie | 24 / 214 (11.2%) | 1.51 (0.89 -2.57 ) | 1.42 (0.86 -2.35 ) | 1.29 (0.62 -2.70 ) |
| **Attended antenatal clinic^2^** |  | P<0.001 | P<0.001 | P<0.001 |
| Yes | 63 / 1942 (3.2 %) | 1 | 1 | 1 |
| No | 97 / 251 (38.6%) | 44.19 (19.23-101.52) | 34.29 (15.79-74.48) | 34.48 (16.70-71.20) |
| **Year (round)** |  | P=0.003 | P=0.001 | P=0.007 |
| 2012-2013 (R23) | 32 / 346 (9.2 %) | 1 | 1 | 1 |
| 2009–2010 (R21/R22) | 57 / 908 (6.3 %) | 0.62 (0.37 -1.02 ) | 0.65 (0.40 -1.07 ) | 0.70 (0.40 -1.23 ) |
| 2007-2008 (R19/R20) | 57 / 733 (7.8 %) | 0.84 (0.51 -1.41 ) | 0.94 (0.57 -1.54 ) | 0.37 (0.20 -0.69 ) |
| 2004-2006 (R16/18) | 54 / 436 (12.4%) | 1.50 (0.88 -2.57 ) | 1.55 (0.92 -2.60 ) | 0.33 (0.15 -0.74 ) |
| 1996 (R8) | 15 / 134 (11.2%) | 1.29 (0.61 -2.73 ) | 1.60 (0.78 -3.29 ) | − |

^1^Final model adjusted for age, antenatal clinic attendance and year. Final model fit using data from 2193 women with complete data surveyed from 2004-2013 (women were not asked about antenatal clinic in 1996 so not included in final model). **^2^**Missing data on marital status for 137 women. Missing data on education for 1 woman, on HIV status for 39 women, and for antenatal clinic attendance for 364 women (134 from round 8 when the question was not asked).
