# Supplementary material for: Adverse pregnancy outcomes in rural Uganda (1996–2013): trends and associated factors from serial cross sectional surveys
Source: BMC Pregnancy Childbirth. 2015 Oct 29;15:279. doi: 10.1186/s12884-015-0708-8 (PMC4627380; doi:10.1186/s12884-015-0708-8)
Supplement: Additional file 3: — Factors associated with stillbirth, among women aged 15–49 years reporting a pregnancy in the past 12 months (1996–2013). (DOCX 15 kb) [file 12884_2015_708_MOESM3_ESM.docx]

**Additional file 3: Factors associated with stillbirth, among women aged 15-49 years reporting a pregnancy in the past 12 months (1996-2013)**

|  | **N stillbirths / N pregnancies (%)** | **Unadjusted OR (95% CI)** | **Age-adjusted OR (95% CI)** |
| --- | --- | --- | --- |
| **Age group** |  | P=0.19 | P=0.19 |
| 20–29 | 38 / 1611 (2.4 %) | 1 | 1 |
| 30–39 | 16 / 799 (2.0 %) | 0.84 (0.44 -1.58 ) | 0.84 (0.44 -1.58 ) |
| 40–49 | 7 / 147 (4.8 %) | 2.12 (0.84 -5.35 ) | 2.12 (0.84 -5.35 ) |
| **Marital status** |  | P=0.88 | P=0.92 |
| Married | 51 / 2034 (2.5 %) | 1 | 1 |
| Divorced/separated/widowed | 5 / 179 (2.8 %) | 1.12 (0.41 -3.07 ) | 1.05 (0.38 -2.89 ) |
| Single (never married) | 4 / 207 (1.9 %) | 0.79 (0.26 -2.35 ) | 0.81 (0.27 -2.43 ) |
| **Education** |  | P=0.73 | P=0.68 |
| None/less than primary | 3 / 169 (1.8 %) | 1 | 1 |
| Incomplete primary | 21 / 1006 (2.1 %) | 1.24 (0.34 -4.59 ) | 1.28 (0.34 -4.77 ) |
| Completed primary | 19 / 654 (2.9 %) | 1.74 (0.46 -6.56 ) | 1.84 (0.48 -6.98 ) |
| Secondary or above | 18 / 727 (2.5 %) | 1.46 (0.39 -5.51 ) | 1.57 (0.41 -6.00 ) |
| **HIV serostatus** |  | P=0.99 | P=0.99 |
| Negative | 55 / 2304 (2.4 %) | 1 | 1 |
| Positivie | 5 / 214 (2.3 %) | 1.00 (0.36 -2.73 ) | 1.01 (0.37 -2.75 ) |
| **Attended antenatal clinic** |  | P=0.39 | P=0.27 |
| Yes | 49 / 1942 (2.5 %) | 1 | 1 |
| No | 4 / 251 (1.6 %) | 0.63 (0.22 -1.81 ) | 0.57 (0.20 -1.66 ) |
| **Year (round)** |  | P=0.12 | P=0.08 |
| 2012-2013 (R23) | 5 / 346 (1.4 %) | 1 | 1 |
| 2009–2010 (R21/R22) | 18 / 908 (2.0 %) | 1.37 (0.48 -3.92 ) | 1.37 (0.48 -3.93 ) |
| 2007-2008 (R19/R20) | 26 / 733 (3.5 %) | 2.72 (0.97 -7.66 ) | 2.76 (0.98 -7.79 ) |
| 2004-2006 (R16/18) | 11 / 436 (2.5 %) | 1.73 (0.56 -5.37 ) | 1.71 (0.55 -5.33 ) |
| 1996 (R8) | 1 / 134 (0.7 %) | 0.51 (0.05 -4.74 ) | 0.53 (0.06 -4.92 ) |
